# The Application of Digital Pathology to Improve Accuracy in Glomerular Enumeration in Renal Biopsies

**DOI:** 10.1371/journal.pone.0156441

**Published:** 2016-06-16

**Authors:** Avi Z. Rosenberg, Matthew Palmer, Lino Merlino, Jonathan P. Troost, Adil Gasim, Serena Bagnasco, Carmen Avila-Casado, Duncan Johnstone, Jeffrey B. Hodgin, Catherine Conway, Brenda W. Gillespie, Cynthia C. Nast, Laura Barisoni, Stephen M. Hewitt

**Affiliations:** 1 Department of Pathology, Children’s National Medical Center, Washington, DC, United States of America; 2 National Institute of Digestive Diseases and Kidney, National Institutes of Health, Bethesda, MD, United States of America; 3 Department of Pathology, University of Pennsylvania, Philadelphia, PA, United States of America; 4 Department of Pathology, University of Miami, Miami, FL, United States of America; 5 Department of Pediatrics, Division of Pediatric Nephrology, University of Michigan, Ann Arbor, MI, United States of America; 6 Department of Pathology, University of North Carolina, Chapel Hill, NC, United States of America; 7 Department of Pathology, The Johns Hopkins School of Medicine, Baltimore, MD, United States of America; 8 Department of Pathology, Toronto General Hospital, Toronto, Ontario, Canada; 9 Department of Medicine, Section of Nephrology, Hypertension and Kidney Transplantation Temple University, Philadelphia, PA, United States of America; 10 Department of Pathology, University of Michigan, Ann Arbor, MI, United States of America; 11 Laboratory of Pathology, Center for Cancer Research, National Cancer Institute, National Institutes of Health, Bethesda, MD, United States of America; 12 Biostatistics Department, School of Public Health, University of Michigan, Ann Arbor, MI, United States of America; 13 Department of Pathology, Cedars-Sinai Medical Center, Los Angeles, CA, United States of America; Institute of Bioengineering and Nanotechnology, SINGAPORE

## Abstract

**Background:**

In renal biopsy reporting, quantitative measurements, such as glomerular number and percentage of globally sclerotic glomeruli, is central to diagnostic accuracy and prognosis. The aim of this study is to determine the number of glomeruli and percent globally sclerotic in renal biopsies by means of registration of serial tissue sections and manual enumeration, compared to the numbers in pathology reports from routine light microscopic assessment.

**Design:**

We reviewed 277 biopsies from the Nephrotic Syndrome Study Network (NEPTUNE) digital pathology repository, enumerating 9,379 glomeruli by means of whole slide imaging. Glomerular number and the percentage of globally sclerotic glomeruli are values routinely recorded in the official renal biopsy pathology report from the 25 participating centers. Two general trends in reporting were noted: total number per biopsy or average number per level/section. Both of these approaches were assessed for their accuracy in comparison to the analogous numbers of annotated glomeruli on WSI.

**Results:**

The number of glomeruli annotated was consistently higher than those reported (p<0.001); this difference was proportional to the number of glomeruli. In contrast, percent globally sclerotic were similar when calculated on total glomeruli, but greater in FSGS when calculated on average number of glomeruli (p<0.01). The difference in percent globally sclerotic between annotated and those recorded in pathology reports was significant when global sclerosis is greater than 40%.

**Conclusions:**

Although glass slides were not available for direct comparison to whole slide image annotation, this study indicates that routine manual light microscopy assessment of number of glomeruli is inaccurate, and the magnitude of this error is proportional to the total number of glomeruli.

## Introduction

The morphologic assessment of renal biopsies is a well-established practice and provides important diagnostic and prognostic information. Standardized assessment and reporting for these specimens is essential [1]. Although most renal biopsy diagnoses are dependent on qualitative features, semi-quantitative metrics are routinely applied for prognostic measures and treatment decisions. Semi-quantitative approaches were first introduced in lupus nephritis and emphasized the detailed evaluation of all lesions in glomerular and tubulointerstitial compartments [2–4]. Subsequently, similar approaches to systematic semi-quantitative biopsy interpretation have been applied to other renal diseases [5–9]. Thus, accuracy in reporting quantitative and semiquantitative values is emphasized by consensus papers for routine renal pathology reporting [1, 10]. Renal pathologists report glomerular numbers in renal biopsies utilizing various approaches, including a) attempting assessment of the total number of glomeruli by looking at all sections available, b) averaging the number of glomeruli per section either counting all glomeruli in each levels as individual glomeruli, or by using the level with the most and the least, c) providing a range using the sections with the least and the most glomeruli. It is, however, appreciated that there are technical limitations independent of the individual pathologists experience and accuracy.

The Nephrotic Syndrome Study Network (NEPTUNE) implemented a digital pathology repository (DPR) that includes whole slide images (WSI) of all kidney biopsy levels available for each case and a copy of the de-identified pathology report [11]. The NEPTUNE digital pathology protocol (NDPP), by application of glomerular annotation in discontinuous but sequential sections, sought to standardize reporting metrics by refining accuracy. The annotation and enumeration of glomeruli in all available WSI sections enables not only the estimation of the overall number of glomeruli, but also of affected glomeruli by any parameter of choice [12].

Here, we applied WSI and annotation software to evaluate the most basic quantitative metrics: total number of glomeruli and number/percentage of globally sclerotic glomeruli. Additionally, by comparing the digital quantitative analysis to the manual light microscopy (LM) analysis as routinely reported, we test its potential value in improving clinical practice.

## Methods

### NEPTUNE DPR and case selection

Renal biopsies are an enrollment criteria of the NEPTUNE study (ClinicalTrials.gov Identifier: NCT01240564). All patients were consented at enrollment, and the study was approved by Institutional Review Boards of all participating institutions (http://www.rarediseasesnetwork.org/cms/neptune).

**Fig 1:**
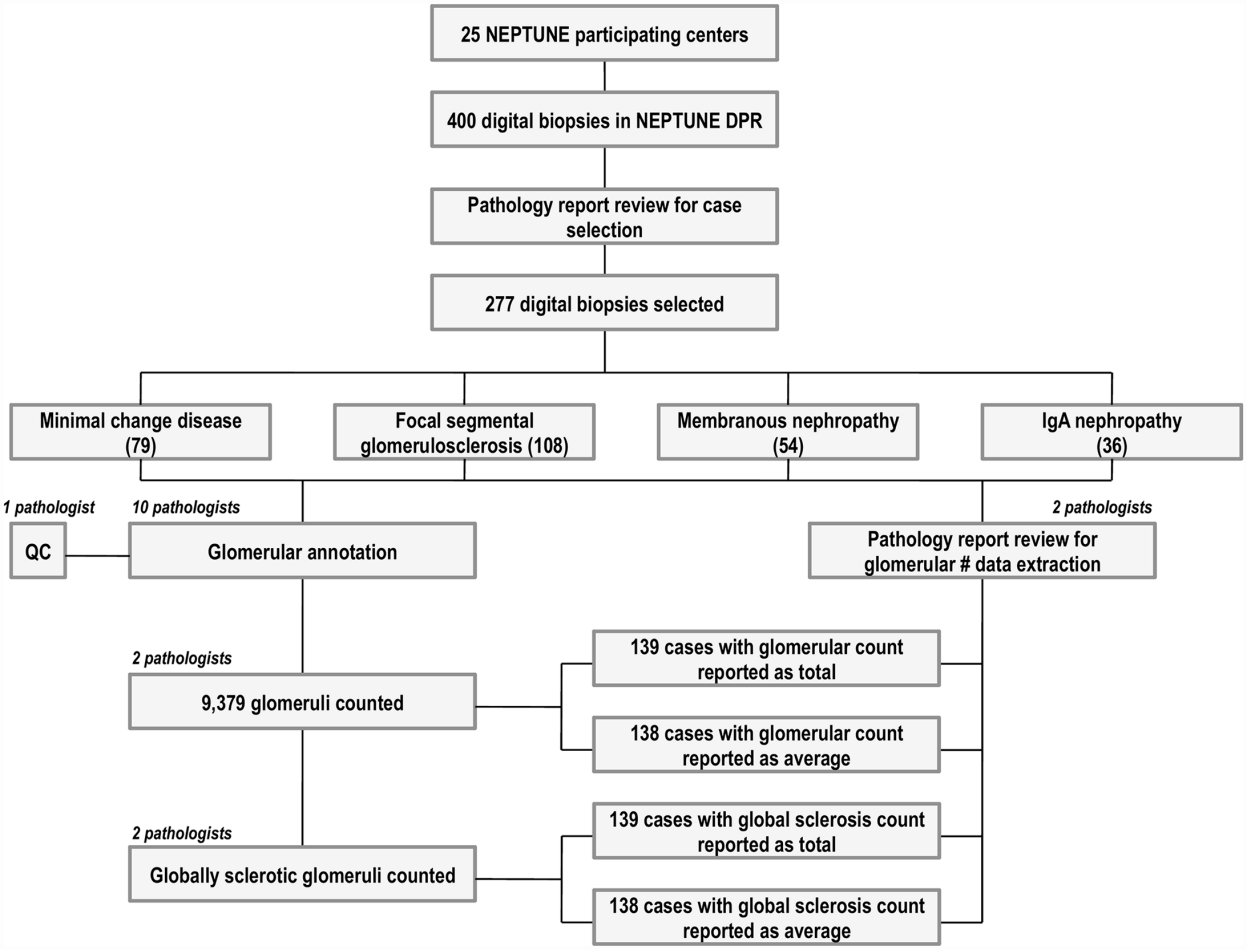
Summary of workflow for case selection and annotation using the NEPTUNE Digital Pathology repository.

According to the NEPTUNE protocol, all renal biopsies are digitized and together with the de-identified PDF of the pathology report, stored in the NEPTUNE DPR [11, 13]. From a total of 392 digital renal biopsies, 317 cases with a diagnosis of minimal change disease (MCD), focal and segmental glomerulosclerosis (FSGS), membranous glomerulopathy (MN), and IgA nephropathy (IgAN) were eligible for inclusion in this study. Other glomerular diseases were excluded. The pdf of the pathology report was reviewed by two pathologists to determine whether the number of glomeruli and the number of globally sclerotic glomeruli (GS) present in the biopsy was documented. Cases where the pathology report was missing the glomerular number assessment (40/317), either as a total number of glomeruli per biopsy as determined by an assessment across all histology sections, or as an average number of glomeruli per histology level, were excluded. A total of 277/317 cases were selected, distributed across the following diagnosis: 79 MCD, 108 FSGS, 54 MN, and 36 IgAN (Fig 1), all of which contain contained documentation of the the assessment of GS. No glass slides were available for re-review as part of this study.

### Glomerular counting

**Fig 2:**
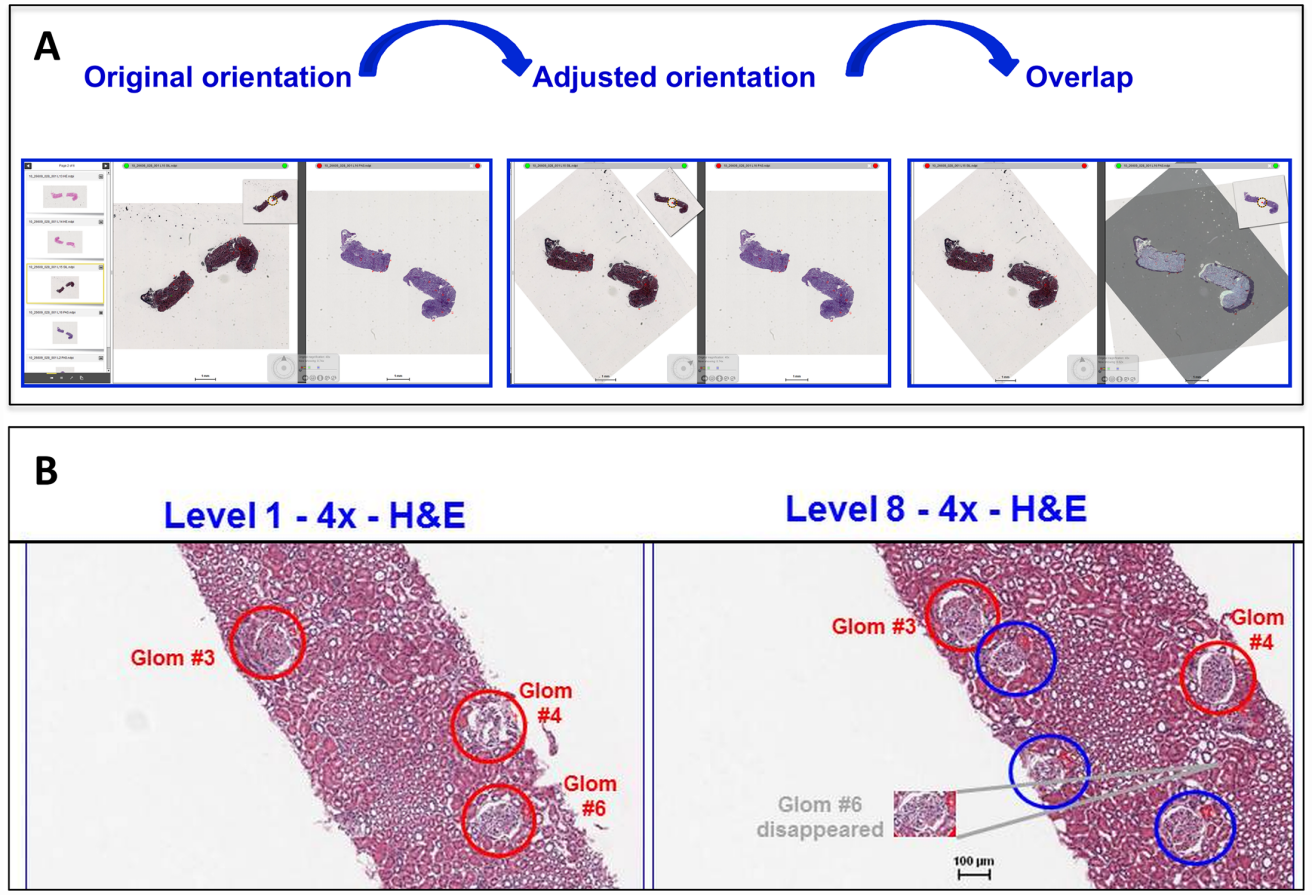
Digital review and annotation of whole slide images in the NEPTUNE digital pathology repository. A) Example of multiple levels visualized at the same time. The software allows overlapping and orientation of the sections to facilitate multilevel reconstruction and representation of the biopsy levels. B) Digital annotation of whole slide images. Two levels are shown in alignment. Glomeruli in the left panel are annotated in red. In a subsequent level (right panel), two of the red-annotated glomeruli are still present (3 and 4), while one has disappeared (6). Three additional glomeruli, including one adjacent to glomerulus 3 and 6 have appeared in the deeper level (annotated in blue).

To test the value of annotation for glomerular enumeration and counting, ten pathologists annotated glomeruli in all WSI levels available by remotely accessing the 277 cases stored in the NEPTUNE DPR (Fig 1). As previously described, annotation of glomeruli was achieved by visualizing and aligning up to 4 WSI simultaneously (DIH, Leica, Dublin IR) and enumerating each glomerulus with a unique number that was maintained in all levels examined [11]. (Fig 2) Following initial annotation, each case underwent quality control review for accuracy of annotation by a different pathologist. Two of the ten pathologists independently retrieved the total number of annotated glomeruli in all 277 cases by remotely accessing each biopsy WSI section/level in the NEPTUNE DPR and recording the highest number used for glomerular annotation.

To investigate concordance between number of glomeruli counted on annotated WSI and number of glomeruli reported in the anonymized pathology reports, reported glomerular count was also recorded by two pathologists either as the absolute total number of glomeruli (139 cases) or the average of the number of glomeruli per histology level (138 cases). The number of annotated glomeruli was then compared to the number of reported glomeruli as follow:

a) for the 139 cases where the total number of glomeruli were recorded in the pathology report, the total number of reported glomeruli per biopsy was compared to the total number of annotated glomeruli counted on the corresponding WSI; b) for the 138 cases where the average number of glomeruli per histology level was reported, this average was compared to the average number of annotated glomeruli per WSI level per biopsy, calculated by dividing the total number of annotated glomeruli per number of WSI levels in each case.

To investigate the impact of annotation on assessment of globally sclerotic glomeruli, two pathologists counted the annotated GS by remotely accessing the NEPTUNE DPR and each biopsy WSI section/level, and recorded the total number or average number of reported GS by reviewing the report pdf. The number and percentage of annotated GS were compared with those recorded in the pathology report. Discrepancy for GS between the two pathologists were resolved by webinar consensus review. The percentages of annotated globally sclerotic glomeruli from WSI and from the pathology reports were calculated using annotated (total or average) and reported number of glomeruli, respectively.

### Statistical methods

Descriptive statistics including mean, range, median and interquartile range (IQR). Wilcoxon signed rank tests were used to test the difference in total number and number of globally sclerotic glomeruli from annotated WSI versus given in pathology reports. The Wilcoxon test was used due to non-normality of the paired differences of annotated versus reported glomeruli. The analysis was performed separately for cases with pathology reports giving total versus average numbers of glomeruli, and presented both overall and with stratification by diagnosis (FSGS, MCD, MN, and IgANP).

To estimate the differences between annotated and reported counts and percentages with global sclerosis by categories of number of glomeruli or percent globally sclerotic glomeruli, linear regression models were used. The outcomes were the differences between annotated and reported values, and glomeruli categories were the covariates. All analyses were conducted in SAS software V9.4 (SAS Institute Inc., Cary, NC, USA).

## Results

### Assessment of annotated glomeruli on WSI

A total of 9,379 annotated glomeruli were counted, of which 1,322 were classified as globally sclerotic. Across all four diagnostic categories, the average number of glomeruli per biopsy was 33 with a range of 3 to 164. Percent globally sclerotic averaged 16.7%, with a range of 0 to 100.

### Comparison of total number of annotated versus reported glomeruli

**Fig 3:**
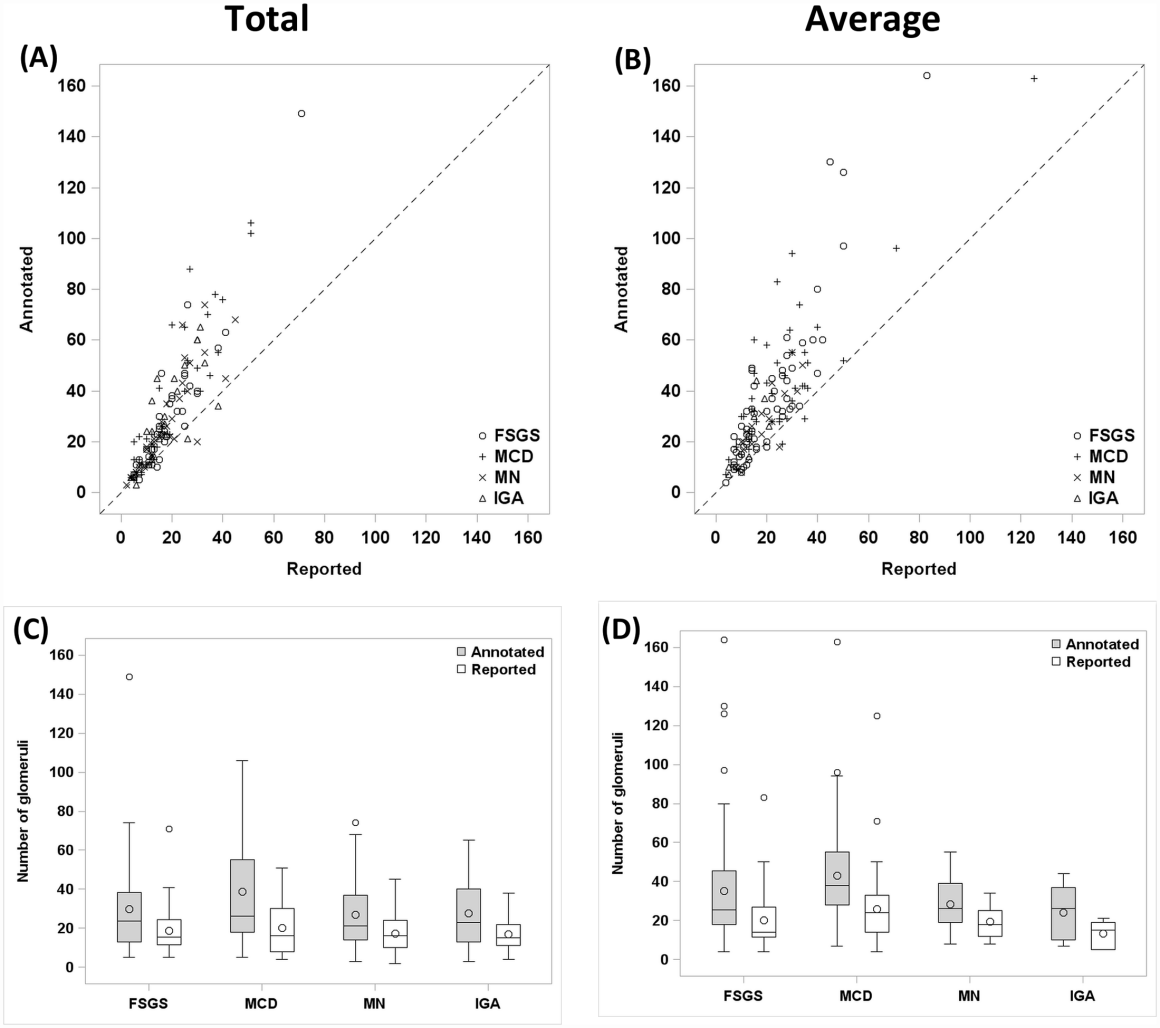
Digital annotation of whole slide images improves accuracy of glomerular enumeration. (A,B) Scatter plot of annotated glomerular number versus reported glomerular number by diagnosis in cases reporting (A) total number of glomeruli or (B) average number of glomeruli per level. Dotted diagonal line shows where annotated = reported, and highlights the increase in number of glomeruli found by annotation. (A: n = 139; B: n = 138). (C,D) Box plot of annotated glomerular number and reported total glomerular number stratified by diagnosis in cases reporting (C) total number of glomeruli (n = 139) or (D) average number of glomeruli (n = 139).

For the 139 cases where the total number of discrete glomeruli was given on the pathology report, the total number of annotated glomeruli per case was higher on average than the total number of glomeruli reported (Annotated: mean glomeruli per case = 30.7; range 3–149; median = 23; IQR 13–43. Reported: mean glomeruli per case = 18.3; range 2–71; median = 16; IQR 10–25; p<0.01) (Table 1). The difference between annotated and reported numbers of glomeruli increased as the number of glomeruli increased, with a ratio approaching 2:1 (Fig 3A). This relationship is also shown in Table 2, where the increase of annotated over reported glomeruli is proportional to the number of reported glomeruli.

**Table 1:** Number of annotated versus reported glomeruli stratified by diagnosis.

Total					Average				
		Reported	Annotated	Significance*			Reported	Annotated	Significance*
**Overall N = 139**	Range	2–71	3–149	<0.01	**Overall N = 138**	Range	4–125	4–164	<0.01
	Mean	18.3	30.7			Mean	21.4	36.0	
**FSGS N = 40**	Range	5–71	5–149	<0.01	**FSGS N = 68**	Range	4–83	4–164	<0.01
	Mean	18.7	29.9			Mean	20.1	35.2	
**MCD N = 33**	Range	4–51	5–106	<0.01	**MCD N = 46**	Range	4–125	7–163	<0.01
	Mean	20.2	38.8			Mean	25.8	42.9	
**MN N = 37**	Range	2–45	3–74	<0.01	**MN N = 17**	Range	8–34	8–55	<0.01
	Mean	17.1	26.8			Mean	19.3	28.2	
**IGA N = 29**	Range	4–38	3–65	<0.01	**IGA N = 7**	Range	5–21	7–44	<0.01
	Mean	16.9	27.6			Mean	13.4	24.0	

Total: n = 139 cases and Average: n = 138 cases.

*Wilcoxon signed rank test

**Table 2:** Difference in glomerular count of number of annotated versus reported glomeruli stratified by number of glomeruli.

Total				Average			
# glom	# cases	Mean Δ (annot—report)	Significance	# glom	# cases	Mean Δ (annot—report)	Significance
**1–10**	37	3.8[0.1,7.5]	0.05	**1–10**	27	5.7[0.6,10.9]	0.03
**11–20**	55	9.0[5.9, 12.0]	<0.01	**11–20**	51	13.5[9.8,17.3]	<0.01
**21–30**	29	20.1[15.9, 24.3]	<0.01	**21–30**	35	15.3[10.7,19.9]	<0.01
**31–40**	12	23.3[16.8,29.9]	<0.01	**31–40**	17	15.8[9.3,22.3]	<0.01
**>40**	6	38.8[29.6,48.1]	<0.01	**>40**	8	46.5[37.0,56.0]	<0.01

Total: n = 139 cases and Average: n = 138 cases.

### Comparison of average number of annotated versus reported glomeruli

Similarly, for the 138 cases where the average number of glomeruli per level was given on the pathology report, the average numbers of annotated glomeruli per level were higher than the average numbers given on pathology reports (Annotated: mean = 36; range 4–164; median = 30; IQR 19–46. Reported: mean = 21.4; range 4–125; median = 16; IQR 12–28; p<0.01)(Table 1). Again, the approximate 2:1 ratio is seen in the scatterplot between annotated and reported average glomeruli (Fig 3B). The increase in the difference with increasing categories of reported glomeruli (Table 2) mirrors the effect seen with total glomeruli above.

### Stratification by center and disease category

Differences in total and average annotated and reported number of glomeruli were maintained across the 25 centers (data not shown) and all disease categories. (Fig 3C and 3D; Table 1)

### Comparison of annotated versus reported globally sclerotic glomeruli

**Fig 4:**
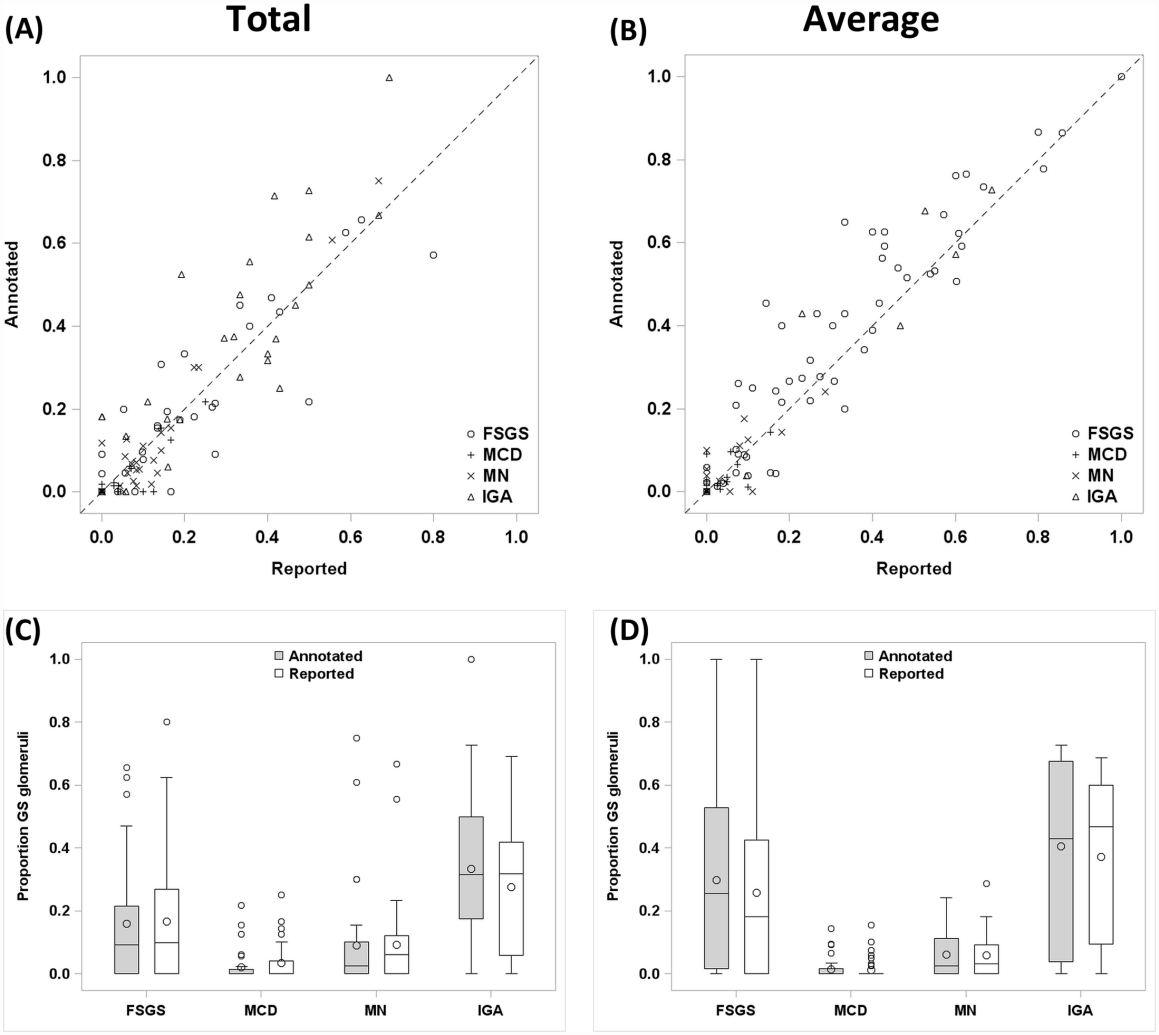
Digital annotation of whole slide images modestly improves accuracy of global glomerular sclerosis enumeration. (A,B) Box plot of annotated percent (%) globally sclerotic glomeruli and reported %globally sclerotic glomeruli in cases reporting (A) total number of glomeruli (n = 139) or (B) average number of glomeruli (n = 138). (C,D) Box plot of difference between number of annotated and reported % globally sclerotic glomeruli stratified by % globally sclerotic glomeruli in cases reporting (C) total number of glomeruli (n = 139) or (D) average number of glomeruli (n = 138).

In the 139 cases where the percentage of globally sclerotic glomeruli was obtained from the reported total number of glomeruli, there was no significant difference in the mean globally sclerotic glomeruli percentage determined on annotated WSI (14.4%) and the pathology reports (13.7%) (Table 3, Fig 4A). In contrast, in cases reporting an average glomerular number (138 cases), annotation yielded a greater mean globally sclerotic glomeruli percentage compared with the corresponding pathology reports (17.9% vs 15.8%, p<0.01) (Table 3, Fig 4B). When cases were stratified by disease (Fig 4C and 4D; Table 3), this discrepancy was statistically significant only in FSGS (29.8% vs 25.8%, p = <0.01). While the overall discrepancy was small in the percentage of globally sclerotic glomeruli between annotated WSI and pathology reports, in cases with > 40% globally sclerotic glomeruli there was a significant underestimation in the biopsy reports by both total and average methodologies (p < 0.01) (Fig 4A and 4B; Table 4); this effect was evident in FSGS and IGA, particularly for the biopsy reports using average glomeruli (Fig 4C and 4D).

**Table 3:** Number of annotated versus reported percent globally sclerotic (%GS) stratified by diagnosis.

Total					Average				
		Reported %GS	Annotated %GS	Significance*			Reported %GS	Annotated %GS	Significance*
**Overall N = 139**	Range	0–80	0–100	0.71	**Overall N = 138**	Range	0–100	0–100	<0.01
	Mean	13.7	14.4			Mean	15.8	17.9	
**FSGS N = 40**	Range	0–80	0–65.6	0.99	**FSGS N = 68**	Range	0–100	0–100	<0.01
	Mean	16.6	16.0			Mean	25.8	29.8	
**MCD N = 33**	Range	0–25	0–21.7	<0.01	**MCD N = 46**	Range	0–15.3	0–14.3	0.81
	Mean	3.3	2.1			Mean	1.2	1.3	
**MN N = 37**	Range	0–66.7	0–75	0.65	**MN N = 17**	Range	0–28.6	0–24.1	0.91
	Mean	9.1	8.8			Mean	5.8	6.1	
**IGA N = 29**	Range	0–69.2	0–100	0.02	**IGA N = 7**	Range	0–68.8	0–72.7	0.69
	Mean	27.4	33.3			Mean	37.2	40.6	

Total: n = 139 cases and Average: n = 138 cases.

*Wilcoxon signed rank test

**Table 4:** Difference in %GS glomeruli between annotated versus reported stratified by number of glomeruli.

Total				Average			
%GS	# cases	Mean Δ (annot—report)	Significance	%GS	# cases	Mean Δ (annot—report)	Significance
**0–19%**	103	-0.80[-22.5,0.1]	0.30	**0–19%**	92	-0.20 [-1.4, 1.1]	0.77
**20–39%**	17	-0.24[-38.4, 3.4]	0.90	**20–39%**	15	3.0 [-0.2, 6.2]	0.07
**40–59%**	10	6.5[1.8,11.2]	<0.01	**40–59%**	17	7.5 [4.5, 10.6]	<0.01
**60–100%**	9	12.8[7.7,17.9]	<0.01	**60–100%**	14	10.3 [7.0, 13.7]	<0.01

Total: n = 139 cases and Average: n = 138 cases.

## Discussion

Glomerular counting is a fundamental metric for renal biopsy providing the denominator for glomerular involvement in primary and secondary kidney diseases. In this study we evaluated the NEPTUNE protocol for glomerular annotation to provide a standardized and reliable estimate of number of glomeruli. Although Whole Slide Imaging is not approved for primary diagnosis in routine pathologic practice in the US [14] it is an enabling technology for the development and validation of evaluation of information gained from light microscopy. WSI with and without annotation has been demonstrated to enhance reproducibility of pathology assessment compared to glass slide review [15–17].

The application of digital annotation facilitates accuracy for renal pathology metric that cannot be achieved by conventional LM. The accuracy of determining the number of glomeruli in a given biopsy by LM is limited by visual-spatial memory of the pathologist across multiple histologic levels. This issue is resolved by the digital software allowing visualization and alignment of multiple levels at the same time, facilitating identification of the same or different glomeruli thru the biopsy. Thus, we found significant differences between the number of glomeruli reported for routine patient care and obtained by LM evaluation, and the number of glomeruli identified by digital annotation. We also demonstrated that discrepancy in glomerular number increases proportionally to the number of glomeruli present within a biopsy, demonstrating the limitation of manual light microscopy to accurately determine the number of glomeruli present. In contrast, the annotated and reported percentages of GS were generally more similar. However, discrepancies were found within disease subgroups and particularly for cases with percent GS over 40%, with annotated values showing higher percent sclerosis in all cases. These data suggest that up to a certain threshold, the discrepancy between annotated and conventional approaches are probably minimally critical for prognostic factors, but may become relevant for biopsies rich in glomeruli and with significant sclerosis. Glomerular density and percentage of GS have been discussed as a risk factor in disease progression [18], IgA [19], risk of progression in membranous glomerulonephritis [20] and obesity-related glomerulopathy [21]. Thus, accurate assessment with improved numerators and denominators is essential in improving prognostication.

Accurate glomerular counting is fundamental to determining biopsy adequacy and improved prognostication. It is anticipated that with digital enumeration of glomeruli, the binomial distribution of abnormal glomeruli (i.e., with segmental lesions), and thus the number of glomeruli needed for accurate sampling, will be greater than previously suggested [22].

Although the ultimate proof of the increased accuracy of digital annotation versus conventional light microscopy would be provided by direct comparison of glomerular number estimate on glass slide and annotated WSI, glass slides were not available and we had to rely on values extracted from the pathology report.

Our study demonstrates the limits of the conventional approach to microscopic examination of tissue in tasks that require enumeration and integration across multiple tissue sections. We have carried out this study in the context of glomerular disease that is a part of the NEPTUNE protocol. The data demonstrates that low frequency events can be accounted for with relative accuracy, as demonstrated by both the overall glomerular enumeration as well as percent global sclerosis data. However, as the number of events increases, the inaccuracy of the estimation increases. With the glomerular number, this reached a level of 2:1 when WSI and manual microscopy are compared. For percent glomerular sclerosis, inaccurate determinations reached statistical significance at 40%GS. These findings suggest that quantization of the histopathology of glomerular disease by an intensive approach, utilizing WSI and piecemeal evaluation of individual glomerular profiles will result in new insights to disease classification, prognostic markers, and potential markers of response to therapy.

These findings suggest the accuracy of detection is object specific, and that findings for one object may not generalize to other objects. Although this study was performed within the limited context of primary glomerular disorders, the findings have broad implications for many observer-based task where integration across image planes is required. The evaluation of renal biopsies for other disorders, including diabetes, SLE and transplant are obvious fields of inquiry, as are renal biopsies of medical conditions of the liver, where portal triads and other features are routinely enumerated. The overall implications are that there are some conventional approaches that may be enhanced by the adoption of computer-aided diagnostic tools. Manual review and enumeration of renal biopsies with current tools is time consuming and complex, however the development of tools to aid the reviewer in a value-added strategy are underway.
